# Interleukin-6-dependent influence of nociceptive sensory neurons on antigen-induced arthritis

**DOI:** 10.1186/s13075-015-0858-0

**Published:** 2015-11-21

**Authors:** Matthias Ebbinghaus, Gisela Segond von Banchet, Julia Massier, Mieczyslaw Gajda, Rolf Bräuer, Michaela Kress, Hans-Georg Schaible

**Affiliations:** Institute of Physiology I/Neurophysiology, Jena University Hospital - Friedrich Schiller University Jena, Teichgraben 8, 07740 Jena, Germany; Institute of Pathology, Jena University Hospital - Friedrich Schiller University Jena, Ziegelmühlenweg 1, 07740 Jena, Germany; Division of Physiology, Department of Physiology and Medical Physics, Innsbruck Medical University, Fritz-Pregl-Strasse 3, 6020 Innsbruck, Austria

**Keywords:** Neurogenic inflammation, Antigen-induced arthritis, CGRP, Interleukin-6, Dorsal root ganglion neurons

## Abstract

**Introduction:**

Interleukin-6 (IL-6) is an important mediator of inflammation. In addition to cells involved in inflammation, sensory nociceptive neurons express the IL-6 signal-transducer glycoprotein 130 (gp130). These neurons are not only involved in pain generation but also produce neurogenic inflammation by release of neuropeptides such as calcitonin gene-related peptide (CGRP). Whether IL-6 activation of sensory neurons contributes to the induction of inflammation is unknown. This study explored whether the action of IL-6 on sensory neurons plays a role in the generation of neurogenic inflammation and arthritis induction.

**Methods:**

In SNS-gp130^−/−^ mice lacking gp130 selectively in sensory neurons and appropriate control littermates (SNS-gp130^flox/flox^), we induced antigen-induced arthritis (AIA), and assessed swelling, histopathological arthritis scores, pain scores, expression of CGRP in sensory neurons, serum concentrations of CGRP and cytokines, and the cytokine release from single cell suspensions from lymph nodes and spleens. In wild-type mice CGRP release was determined during development of AIA and, in cultured sensory neurons, upon IL-6 stimulation.

**Results:**

Compared to SNS-gp130^flox/flox^ mice SNS-gp130^−/−^ mice showed significantly weaker initial swelling, reduced serum concentrations of CGRP, IL-6, and IL-2, no inflammation-evoked upregulation of CGRP in sensory neurons, but similar histopathological arthritis scores during AIA. During the initial swelling phase of AIA, CGRP was significantly increased in the serum, knee and spleen. In vitro, IL-6 augmented the release of CGRP from cultured sensory neurons. Upon antigen-specific restimulation lymphocytes from SNS-gp130^−/−^ mice released more interleukin-17 and interferon-γ than lymphocytes from SNS-gp130^flox/flox^ mice. In naive lymphocytes from SNS-gp130^flox/flox^ and SNS-gp130^−/−^ mice CGRP reduced the release of IL-2 (a cytokine which inhibits the release of interleukin-17 and interferon-γ).

**Conclusions:**

IL-6 signaling in sensory neurons plays a role in the expression of arthritis. Selective deletion of gp130 signaling in sensory neurons reduces the swelling of the joint (most likely by reducing neurogenic inflammation) but increases some proinflammatory systemic cellular responses such as the release of interleukin-17 and interferon-γ from lymphocytes upon antigen-specific restimulation. Thus IL-6 signaling in sensory neurons is not only involved in pain generation but also in the coordination of the inflammatory response.

## Introduction

The proinflammatory cytokine interleukin-6 (IL-6) is a key player in innate immunity and joint inflammation including human rheumatoid arthritis (RA) [1–4], and therefore it has been developed into a target of disease-modifying drugs. Tocilizumab (an IL-6 receptor-specific antibody) and tofacitinib (a JAK inhibitor) have been approved for RA therapy, and sirukumab (an IL-6-specific antibody) and sarilumab (an IL-6 receptor-specific antibody) are promising compounds in phase III clinical development [4].

IL-6 binds to membrane-bound IL-6 receptor (IL-6R) or to soluble IL-6 receptor (sIL-6R), which is present in the plasma. Because IL-6/sIL-6R complexes can bind to cells without membrane-bound IL-6R (a process called transsignaling [4]), IL-6 can act on many different cell types such as immune cells, osteoclasts, and others even if they do not express membrane-bound IL-6R [2, 4]. Essential for the activation is the transmembrane signal-transducing subunit glycoprotein 130 (gp130) [3, 4], which is ubiquitously expressed. While inhibition of IL-6 signaling significantly attenuates murine arthritis [5–7], hyperactive gp130 signaling exacerbates inflammation [8].

IL-6 acts also on nociceptive neurons and is therefore regarded as a putative pain mediator [9–11]. In fact, dorsal root ganglion (DRG) neurons, the cell bodies of sensory neurons, express the signal transducer gp130 [11, 12], and gp130 expressed in nociceptive sensory neurons is now considered a key regulator of the induction and maintenance of mechanical hyperalgesia [13–15]. Upon activation, a proportion of the DRG neurons can also release neuropeptides such as substance P and calcitonin gene-related peptide (CGRP) into the tissue and cause a so-called neurogenic inflammation [16–18]. Remarkably, the levels of these neuropeptides and IL-6 were shown to be enhanced in serum and synovial fluid of RA and osteoarthritis patients [19]. An intriguing hypothesis is, therefore, that IL-6 causes enhanced release of neuropeptides from sensory neurons and that part of the proinflammatory effect of IL-6 is produced via the nervous system.

In order to specifically address the role of IL-6 stimulation of sensory neurons, we studied the development of antigen-induced arthritis (AIA) in SNS-gp130^−/−^ mice, which lack the IL-6 signal-transducing receptor subunit gp130 selectively in primary sensory neurons. In SNS-gp130^−/−^ mice the Cre recombinase is under the transcriptional control of the gene of the sodium channel Na_v_1.8, which is selectively expressed in nociceptive sensory neurons [14], and therefore gp130 is selectively deleted in nociceptive primary sensory neurons. For comparison we used their control floxed littermates (SNS-gp130^flox/flox^), in which gp130 remains intact. Furthermore, we tested whether CGRP is released during developing inflammation and whether IL-6 can cause release of CGRP from sensory neurons. We used the model of unilateral AIA in mice.

AIA, originally described by Brackertz et al. [20], is a T cell-mediated joint inflammation whose histopathology in the affected joint shows many similarities to RA. In immunized animals, AIA can be induced after injection of the antigen mBSA into the knee joint as unilateral monoarthritis. Advantages are the precise starting point of arthritis, defined by the intraarticular injection of the antigen into the knee joint, the incidence of 100 %, and the reproducible time course. It shows a transition from an acute stage with strong swelling, dense infiltration of the synovial membrane by granulocytes and exudation of granulocytes into the joint space to a chronic stage with hyperplasia of synovial lining cells, infiltration of the synovial membrane by mononuclear cells, fibrosis of the synovial membrane and the periarticular tissue, and some destruction of cartilage. The unilateral process allows using side-by-side comparison. The AIA model is extensively used for pain research. In animals with AIA the level of IL-6 in serum and synovial fluid is increased and shows a strong correlation with the severity of the disease [21].

We found that SNS-gp130^−/−^ mice show significantly less swelling of the knee joint than SNS-gp130^flox/flox^ mice, and no upregulation of CGRP in sensory neurons as in wild-type and SNS-gp130^flox/flox^ mice indicating that IL-6 effects on sensory neurons contribute to the inflammatory swelling. CGRP is released within the first hours of developing AIA and IL-6 causes release of CGRP from cultured sensory neurons. The serum of SNS-gp130^−/−^ mice at day 3 of AIA exhibited lower concentrations of CGRP, IL-6, and IL-2 than the serum of SNS-gp130^flox/flox^ mice. The antigen-specific-restimulated lymphocytes isolated from lymph nodes and the spleen of SNS-gp130^flox/flox^ mice released significantly more interleukin-17 (IL-17) and interferon-γ (IFNγ) than lympocytes from SNS-gp130^flox/flox^ mice. Thus the loss of neuronal IL-6 signaling also affects the cellular immune response.

## Methods

### Animals

We used 51 C57BL/6 J male/female wild-type (WT) mice (12–14 weeks, 17–22 g, supplied by the Animal Facility of the University Hospital Jena), 26 male/female SNS-gp130^−/−^ mice and 28 male/female control littermates (SNS-gp130^flox/flox^) (8–16 weeks, 20–31 g, bred and genotyped by the Innsbruck Medical University). SNS-gp130^−/−^ mice were generated using the Cre recombinase floxP strategy. Homozygous mice carrying the loxP-flanked (floxed) gp130 allele [22] were mated with mice which express Cre recombinase selectively in sensory neurons using promoter elements of the Na_v_1.8 gene (Scn10a) [23]. The generation of mice at the University of Innsbruck was approved by the Austrian Bundesministerium fuer Wissenschaft und Forschung. All experiments on animals at the University of Jena were approved by the Thüringer Landesamt für Lebensmittelsicherheit and Verbraucherschutz, Abteilung Gesundheitlicher Verbraucherschutz, Veterinärwesen, Pharmazie (registration number No. 02-033/10). At the end of the experiments mice were killed by cervical dislocation under isoflurane anesthesia.

### Arthritis induction and assessment

Mice were immunized at 21 and 14 days before AIA induction with subcutaneous injection of 100 μg of methylated bovine serum albumin (mBSA), the antigen (Sigma-Aldrich, Taufkirchen, Germany), emulgated with 50 μl of complete Freund’s adjuvant (CFA; Sigma-Aldrich), supplemented to 2 mg/ml Mycobacterium tuberculosis, strain H37Ra (Difco, Detroit, MI, USA). Additionally, 5 × 10^8^ heat-inactivated Bordetella pertussis germs (Chiron-Behring, Marburg, Germany) were applied intraperitoneally. For induction of monoarticular AIA 100 μg mBSA in 25 μl 0.9 % NaCl was injected into the right knee joint cavity on day 0. Immunization control animals (IC) received 25 μl 0.9 % NaCl intraarticularly. A flare-up reaction was induced by another intraarticular injection of 100 μg mBSA 21 days after primary arthritis induction. The knee injections were performed under short isoflurane (2.5 %) anesthesia.

Knee swelling was assessed by M. Ebbinghaus by measuring the mediolateral joint diameter using an Oditest vernier caliper (Kroeplin, Schlüchtern, Germany). For histopathology, knee joints were removed, fixed in 4.5 % formalin, decalcified in EDTA, embedded in paraffin and cut into 3 μm frontal sections, which were stained with hematoxylin and eosin (H&E). The pathologist who scored the arthritis (M. Gaida) was unaware of the experimental groups, and arthritis was assessed as previously established [24]. Signs of acute inflammation (infiltration of the synovial membrane by granulocytes and exudation of granulocytes into the joint space), and chronic inflammation (hyperplasia of synovial lining cells, infiltration of the synovial membrane by mononuclear cells, fibrosis of the synovial membrane and the periarticular tissue) were scored: 0: no, 1: mild, 2: moderate, 3: severe changes (+1 if fibrin exudation in the joint space). Cartilage surface defects with cell necrosis were scored: 0: no damage, 1: <5 %, 2: 5–10 %, 3: 11–50 %, and 4: >50 % of the cartilage surface affected. Damage to bone was evaluated: 0: no, 1: mild, 2: medium, 3: severe (extensive area of deep invasive destruction of bone). Scoring was graded in 0.5 steps.

### Pain-related behavior

Secondary mechanical and thermal hyperalgesia at the hindpaws were assessed as an indicator of pain. After accommodation to the device the mechanical pain threshold was assessed with a dynamic plantar esthesiometer (Ugo Basile, Comerio, Italy), which applied increasing pressure at 1 g/s to the paw (cutoff at 10 g). The latency of the elicited leg withdrawal, which reflects the mechanical threshold, was averaged from up to three consecutive stimuli. Two testings before AIA induction defined the baseline (BL). Thermal hypersensitivity was assessed using the Hargreaves plantar test (Ugo Basile) [25]. Three consecutive standardized heat stimuli were applied to the paw to evaluate the mean withdrawal latency (cutoff at 20 s). To quantify the magnitude of hyperalgesia we calculated the area under the curve (AUC) for the values of the right (ipsilateral side of inflammation) and left (contralateral side) paw [26].

### Cytokine analysis

Using standard sandwich enzyme-linked immunosorbent assay (ELISA) procedures [27], cytokines were analyzed in serum and single cell suspensions from inguinal, popliteal, subaortic lymph nodes and spleens, removed at day 3 of AIA, and in cell suspensions from spleens from naïve mice. Primary and biotin-labeled secondary antibodies for IFNγ, IL-2, IL-4, IL-5 and IL-6 were purchased from BD Biosciences (Heidelberg, Germany), antibodies for IL-17 from R&D Systems (Wiesbaden, Germany). For quantification, recombinant cytokines were used as standard.

Because lymphocytes show little basal release of cytokines in vitro, lymphocytes (10^6^ cells/ml) were cultured for 42 h with either 1 μg/ml plate-bound anti-CD3 antibodies (from 145-2C11 hybridoma cell supernatant) for overall T cell receptor stimulation, or with 25 μg/ml mBSA for antigen-specific restimulation. Lymphocytes from each mouse were stimulated separately and the cytokines were measured, cytokine by cytokine, in aliquots of the supernatant from the lymphocytes of each mouse. Each experimental group contained data from the lymphocytes of at least six mice.

### Serum antibody levels

Total immunoglobulin G (IgG) and IgG specific for mBSA were determined in serum, obtained at day 3 of AIA, by ELISA (see [27]). IgG levels are illustrated as the value of absorbance representing readings obtained at 492 nm for total IgG and at 405 nm for the IgG subclasses, respectively.

### CGRP analysis and protein assay

CGRP was quantified in serum, tissue extracts from knee joints, spleens and in supernatants of DRG neuron cultures. The tissues were snap-frozen without further dissection, i.e., the whole organ was used without separating different tissues. Extracts from snap-frozen tissue were prepared in an extraction buffer (pH 3.4) containing protease inhibitors using an ultrasonicator (Covaris, Brighton, UK) for lysis after manual tissue homogenization with liquid nitrogen. Total protein concentration in extracts was determined with a BCA protein assay (Thermo Fisher Scientific, Rockford, IL, USA) according to the manufacturer’s instructions. For CGRP measurement we used an enzyme immunoassay kit (Cusabio, Wuhan, China) according to the manufacturer’s instructions. The intra-assay coefficient of variation (CV) was <8 %, the inter-assay CV was <10 % and the sensitivity limit was 3.9 pg/ml.

### Immunohistochemical labeling of CGRP in DRG sections

DRGs from segments L1–5 from both sides were excised separately, fixed at 4 °C in 4 % paraformaldehyde for 24 h, embedded in paraffin, cut into 5 μm sections, which were dewaxed and autoclaved for 15 min (120 °C, 1 bar). For CGRP labeling we applied overnight at 4 °C a mouse reactive anti-CGRP antibody (1:100; polyclonal against a synthetic rat Tyr-CGRP (23–37) conjugated to gamma globulin developed in goat; Cat. N^o^ BP022, Acris Antibodies, Herford, Germany). Sections were incubated for 2 h in biotinylated rabbit anti-goat antibody (1:200; DAKO, Glostrup, Denmark), then the avidin-biotin peroxidase complex (Vectastatin Elite ABC Kit, Vector Laboratories, Burlingame, CA, USA) was applied for 40 min. Sections were developed with Jenchrom px blue (JenLab, Jena, Germany). In control experiments the primary antibody was omitted or preincubated with 100 μg/ml CGRP (1–37) for 20 min (Bachem, Bubendorf, Germany).

The immunohistochemical labeling was evaluated as previously described [28]. In each second section the proportion of neuronal profiles with CGRP-like immunoreactivity (IR) was determined using a light microscope (Axioplan 2, Zeiss, Jena, Germany) coupled to an image analyzing system (AxioVision, Zeiss). For each mouse the average proportion of labeled neuronal profiles was calculated. At least 100 neuronal profiles with a visible nucleus per mouse were counted.

### CGRP release from cultured DRG neurons

DRGs from WT mice were dissected, incubated in 125 U/ml collagenase type II (Paesel and Lorei, Hanau, Germany), diluted in Ham’s F12 (PAA Laboratories, Piscataway, NJ, USA), for 1 h at 37 °C. After washing, ganglia were placed in 10,000 U/ml Trypsin (Sigma-Aldrich), diluted in Dulbecco’s modified Eagle’s medium (DMEM, Gibco/BRL, Eggenstein-Leopoldshafen, Germany) for 11 min at 37 °C. Neurons were separated by gentle agitation and mechanical treatment with a fire-polished Pasteur pipette. The cell suspension was centrifuged at 500 × g (8 min), finally neurons were resuspended in media consisting of Ham’s F12, 50 μmol/l β-mercaptoethanol (Roth, Karlsruhe, Germany), 10 ng/ml nerve growth factor (Enzo Life Sciences, Lörrach, Germany), 2 mmol/l glutamine (Sigma-Aldrich), 15 % heat inactivated horse serum (PAA Laboratories), 100 U/ml penicillin, 100 μg/ml streptomycin (Gibco/BRL). The cell suspension was plated on poly-L-lysine- (200 μg/ml; Sigma-Aldrich) coated glass coverslips and kept in a humidified incubator gassed with 5 % CO_2_ and air at 37 °C. Cells were fed with 1 ml medium every day.

CGRP release was measured from DRG neurons cultured for 2 days. Basal CGRP production was measured in the presence of culture medium for 20 min. For stimulation we applied either 50 mmol/l KCl alone for 20 min, or KCl with IL-6 or/and soluble IL-6 receptor (sIL6R) (for concentrations see Fig. 6d). Phosphate-buffered saline (PBS), containing 10 % fetal calf serum, was added to culture supernatant aliquots to protect CGRP from degradation. Probes were stored at −20 °C until usage.

### Statistical analysis

Differences were calculated using the two-tailed Students *t* test for unpaired values and the nonparametric Mann-Whitney *U* and Kruskal-Wallis test for CGRP analysis respectively. Arthritis scores and proportions of labeled neurons were compared using analysis of variance (ANOVA) followed by pairwise multiple comparison procedures (Bonferroni, *t* test). The correlation between the histopathological arthritis score and the joint swelling was analyzed using Spearman’s rank correlation coefficient. Differences in cytokine expression against baseline were analyzed using the Wilcoxon’s matched-pairs signed-rank test. Statistical significance was calculated with SPSS software (v.16.0, Chicago, IL, USA) and accepted at *p* < 0.05.

## Results

### Swelling and pain-related behavior in AIA in SNS-gp130^−/−^ and SNS-gp130^flox/flox^ mice

**Fig. 1:**
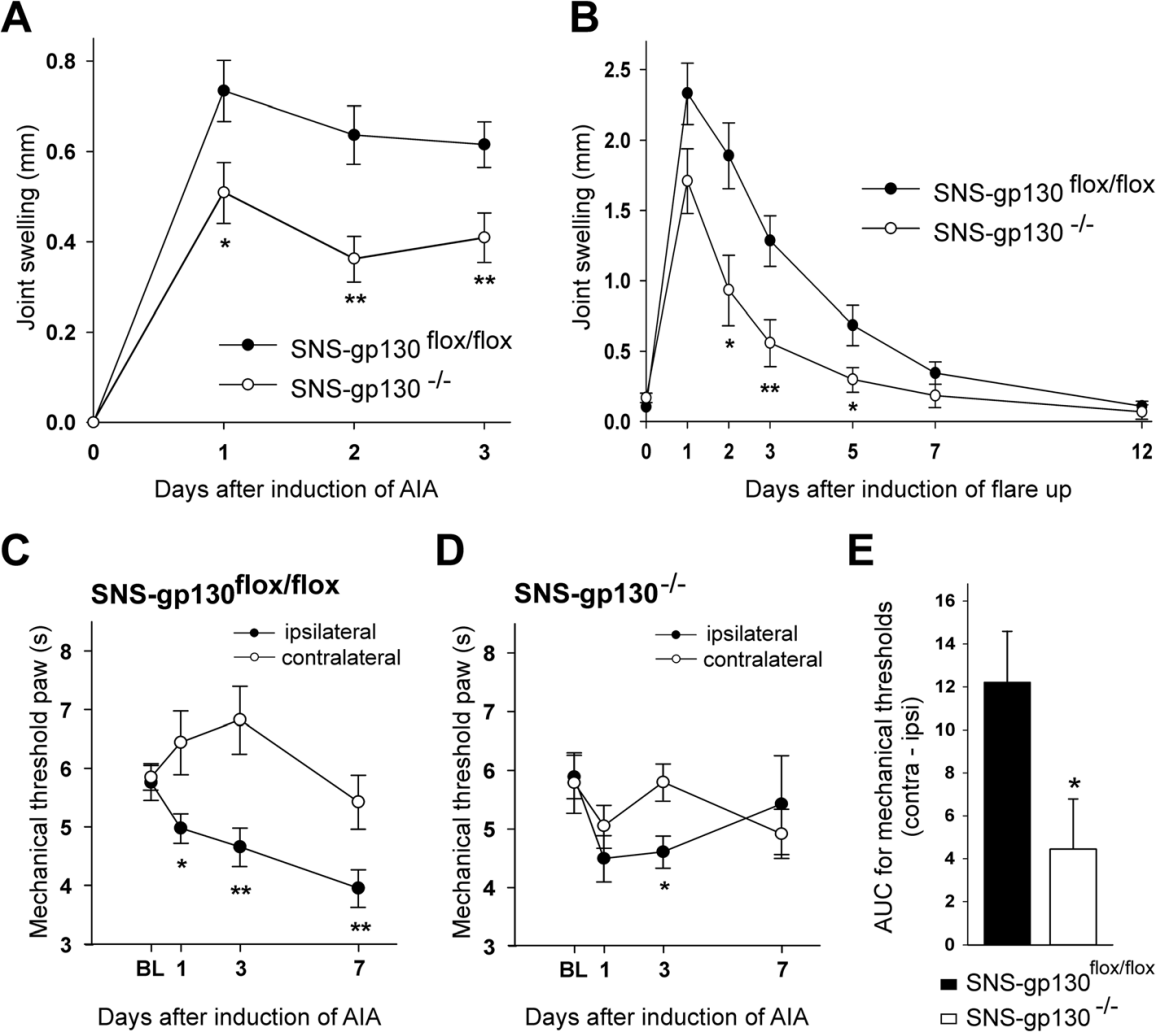
Joint swelling and pain-related behaviors in SNS-gp130^flox/flox^ and SNS-gp130^−/−^ mice after induction of AIA. **a**. In acute primary AIA SNS-gp130^−/−^ mice (*n* = 16) show significantly less joint swelling than SNS-gp130^flox/flox^ mice (*n* = 19) (F[1, 33] = 9.476; *p* = 0.004). **b**. The same effect was observed in the flare-up reaction (induced 21 days after the primary AIA) up to day 7 (F[1, 19] = 8.438; *p* = 0.009; *n* = 10–11 per group). **c**. Reduction of mechanical withdrawal threshold at the paw ipsilateral to the inflamed knee and increase in threshold at the paw contralateral to the inflamed knee in SNS-gp130^flox/flox^ mice (F[1, 11] = 28.298; *p* < 0.001). **d**. No significant differences between the ipsilateral and contralateral paw in SNS-gp130^−/−^ mice after AIA induction (F[1, 13] = 0.713; *p* = 0.414). In c and d: *n* = 6–8 per group. **e**. Areas between the ipsilateral and contralateral curves in c and d. Values are mean ± SEM, *p < 0.05, **p < 0.01. *AIA* antigen-induced arthritis, *AUC* area under the curve, *BL* baseline

Initially we tested whether the deletion of gp130 in sensory neurons impairs the development of AIA. As shown in Fig. 1a, joint swelling was significantly attenuated in SNS-gp130^−/−^ mice compared to swelling in SNS-gp130^flox/flox^ mice. This strong effect of the deletion of neuronal gp130 was also observed in the flare-up reaction (a second acute reaction), which was evoked by a further injection of mBSA into the knee joint at 21 days after the primary AIA induction (Fig. 1b). Differences between both groups of mice disappeared during the transition into the chronic phase of AIA from day 7 on.

We also measured the pain-related behavior (secondary hyperalgesia at the paws) of SNS-gp130^−/−^ and SNS-gp130^flox/flox^ mice before and during development of AIA. In immunized but nonarthritic mice the time to withdrawal of the leg (indicating withdrawal thresholds) to mechanical stimuli onto the right hind paw were not different in SNS-gp130^−/−^ (5.9 ± 0.4 s) and SNS-gp130^flox/flox^ mice (5.8 ± 0.3 s). After AIA induction SNS-gp130^flox/flox^ mice showed a reduction of mechanical threshold for withdrawal at the ipsilateral (right) paw and a slight increase of threshold at the contralateral paw (Fig. 1c, significant difference between both paws, ANOVA: F[1, 11] = 28.298; *p* < 0.001). SNS-gp130^−/−^ mice did not show such a difference (ANOVA: F[1, 13] = 0.713; *p* = 0.414) indicating less mechanical hyperalgesia than in SNS-gp130^flox/flox^ mice (Fig. 1d). Figure 1e compares the areas between the curves displayed in Fig. 1c and d. Testing for thermal hyperalgesia did not show differences between SNS-gp130^flox/flox^ and SNS-gp130^−/−^ mice (data not shown).

### Histopathological scores of AIA in SNS-gp130^−/−^ and SNS-gp130^flox/flox^ mice

**Fig. 2:**
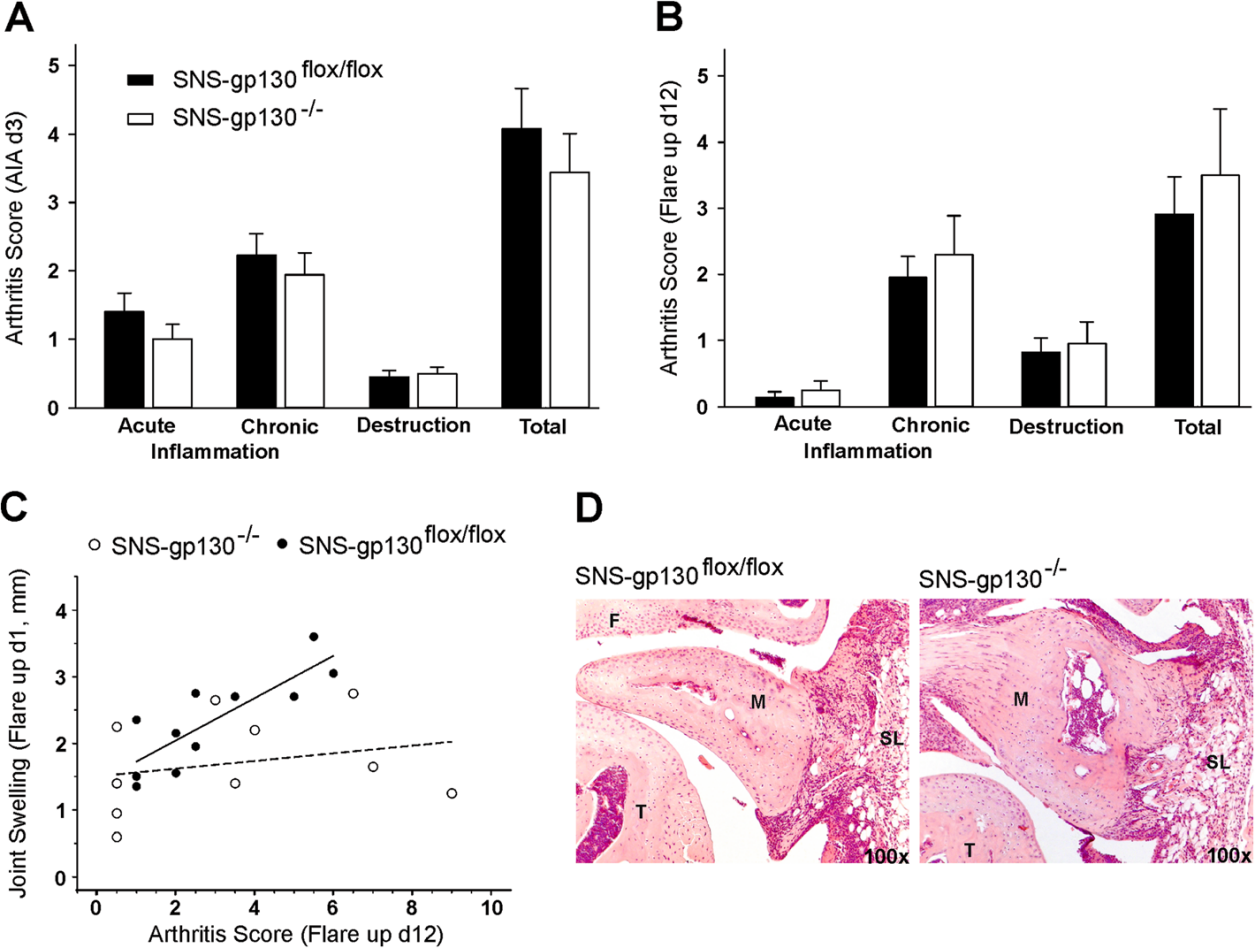
Histopathological scores of AIA in SNS-gp130^−/−^ and SNS-gp130^flox/flox^ mice. **a**. Scores of synovitis and damage to articular cartilage or bone in both groups of mice at day 3 of primary AIA. **b**. Scores of inflammation and destruction in both groups of mice at day 12 of flare-up. Values in a and b are mean ± SEM. **c**. Correlation between joint swelling and histopathological arthritis score in SNS-gp130^flox/flox^ mice (*solid line*, *r* = 0.831, *p* = 0.002) and SNS-gp130^−/−^ mice (*broken line*, *r* = 0.267, *p* = 0.457), *n* = 10–11 per group. **d**. Representative pictures (100×) of H&E-stained knee joint sections from SNS-gp130^flox/flox^ and SNS-gp130^−/−^ mice at day 3 of AIA. *AIA* antigen-induced arthritis, *F* femur, *M* meniscus, *SL* synovial layer, *T* tibia,

Although SNS-gp130^−/−^ mice exhibited significantly less swelling, the histopathological arthritis scores of inflammation and destruction did not show significant differences between SNS-gp130^flox/flox^ and SNS-gp130^−/−^ mice, neither on day 3 of acute AIA (Fig. 2a) nor on day 12 after flare-up (Fig. 2b). However, we noted a striking difference between SNS-gp130^−/−^ and SNS-gp130^flox/flox^ mice in the relationship between swelling and the histologic arthritis score. Figure 2c displays the value of swelling of day 1 of flare-up against the corresponding arthritis score of each mouse. SNS-gp130^flox/flox^ mice showed a strong correlation of swelling and arthritis score (r = 0.831, *p* = 0.002), whereas SNS-gp130^−/−^ mice did not (r = 0.267, *p* = 0.457) indicating that the processes of swelling and cellular inflammatory effects in SNS-gp130^−/−^ mice were not coherent. Figure 2d displays representative H&E-stained knee joint sections.

### Alteration of mediators in the serum and of cellular and humoral immune responses

**Fig. 3:**
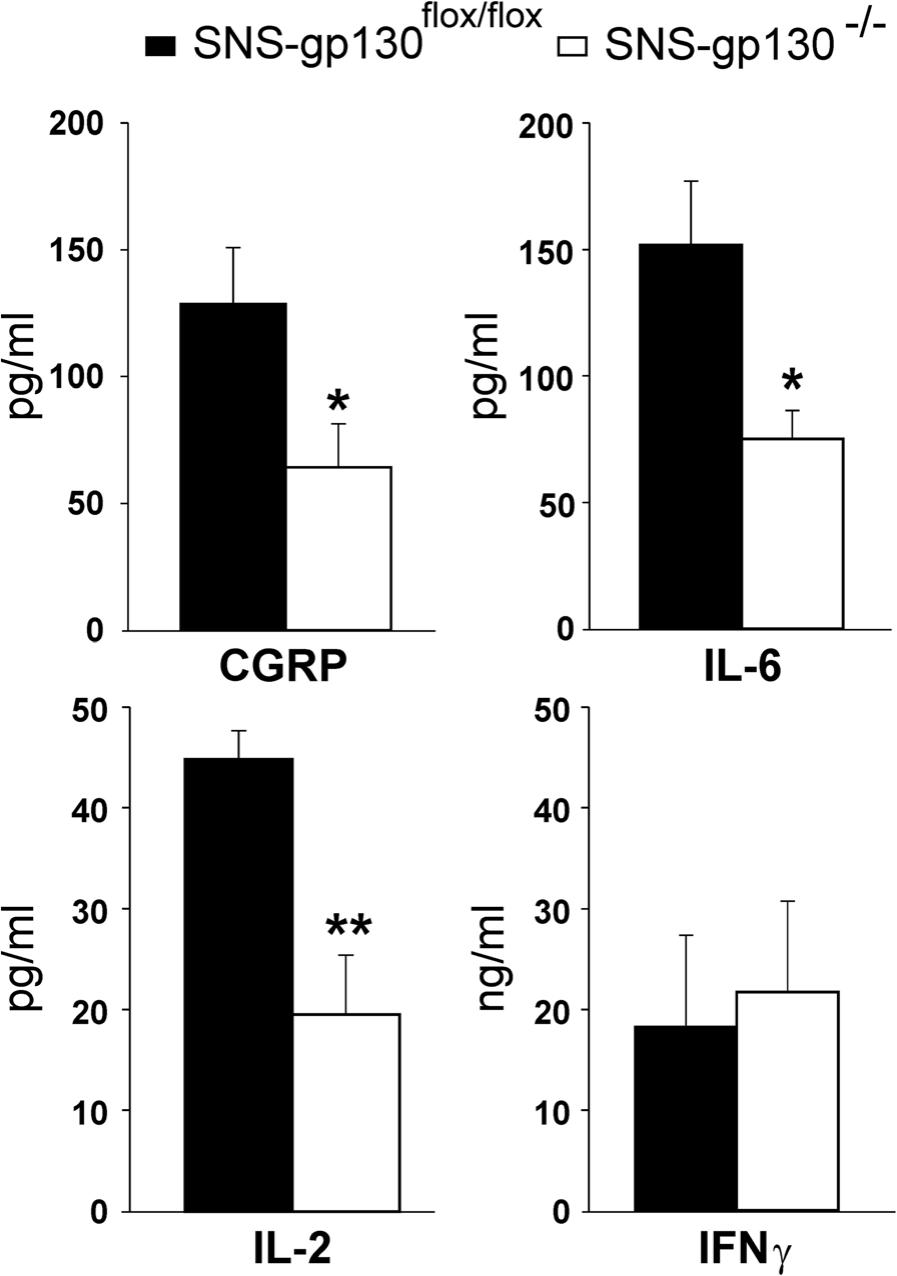
Serum concentrations of CGRP, IL-6, IL-2 and IFNγ in SNS-gp130^flox/flox^ (*black bars*, *n* = 10) and SNS-gp130^−/−^ mice (*white bars*, *n* = 7) on day 3 of AIA. IL-17 was not detectable. Values are mean ± SEM, *p < 0.05, **p < 0.01. *CGRP* calcitonin gene-related peptide, *IFNγ* interferon-γ, *IL* interleukin

**Fig. 4:**
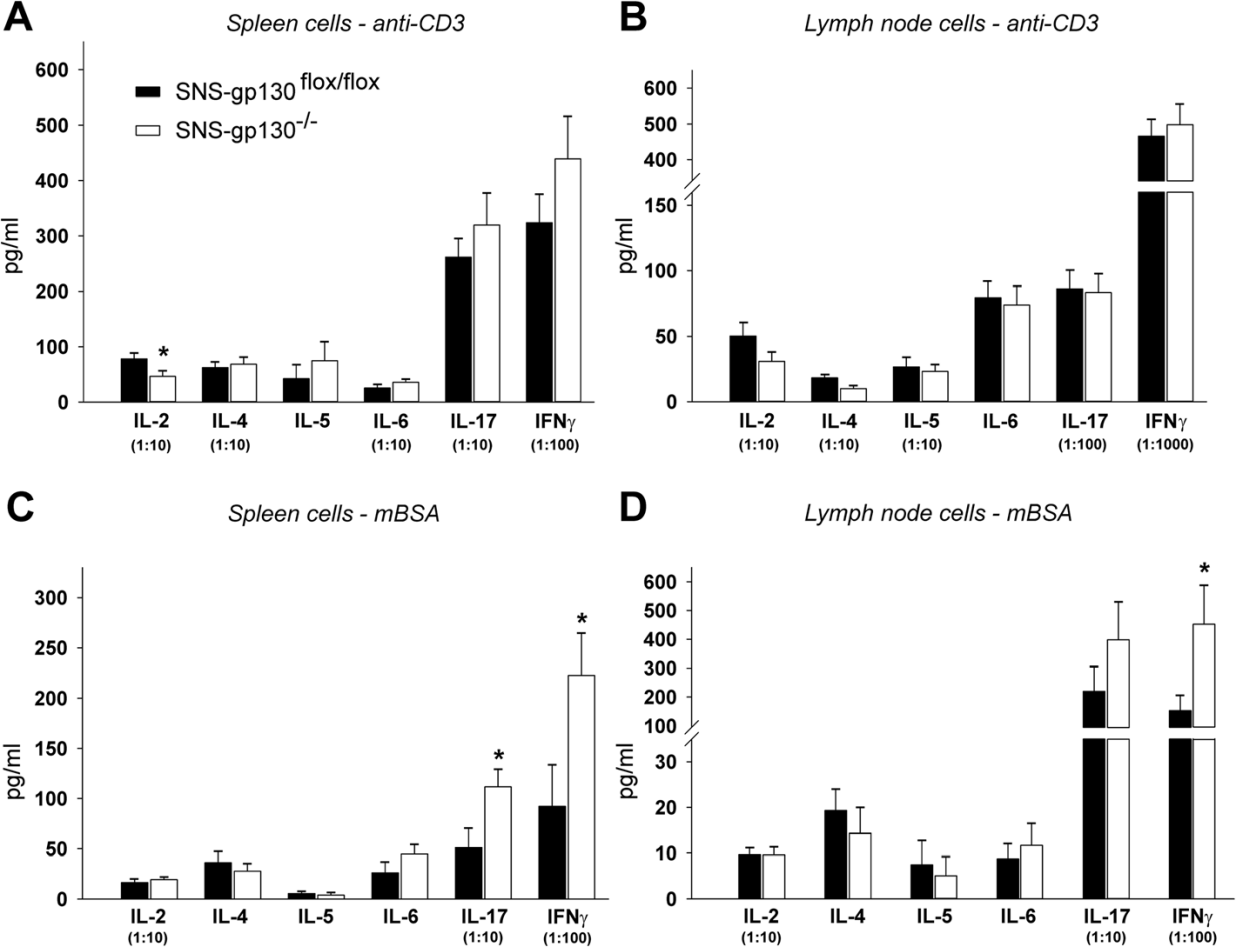
In vitro cytokine expression profiles of lymphocytes isolated on day 3 of AIA from SNS-gp130^flox/flox^ and SNS-gp130^−/−^ mice in vitro. **a**. Cytokine levels in supernatants of anti-CD3-stimulated spleen cells (IL-2, *p* = 0.039). **b**. Cytokine levels in supernatants of anti-CD3-stimulated lymph node cells. **c**. Cytokine levels in supernatants of antigen (mBSA)-specific restimulated spleen cells (IL-17, *p* = 0.035; IFNγ, *p* = 0.034). **d**. Cytokine levels in supernatants of antigen (mBSA)-specific restimulated lymph node cells (IFNγ, *p* = 0.038). Specifications in brackets define the dilution of the cytokine values. Values are mean ± SEM, *n* = 8–10 per group. *IFNγ* interferon-γ, *IL* interleukin, *mBSA* methylated bovine serum albumin

**Fig. 5:**
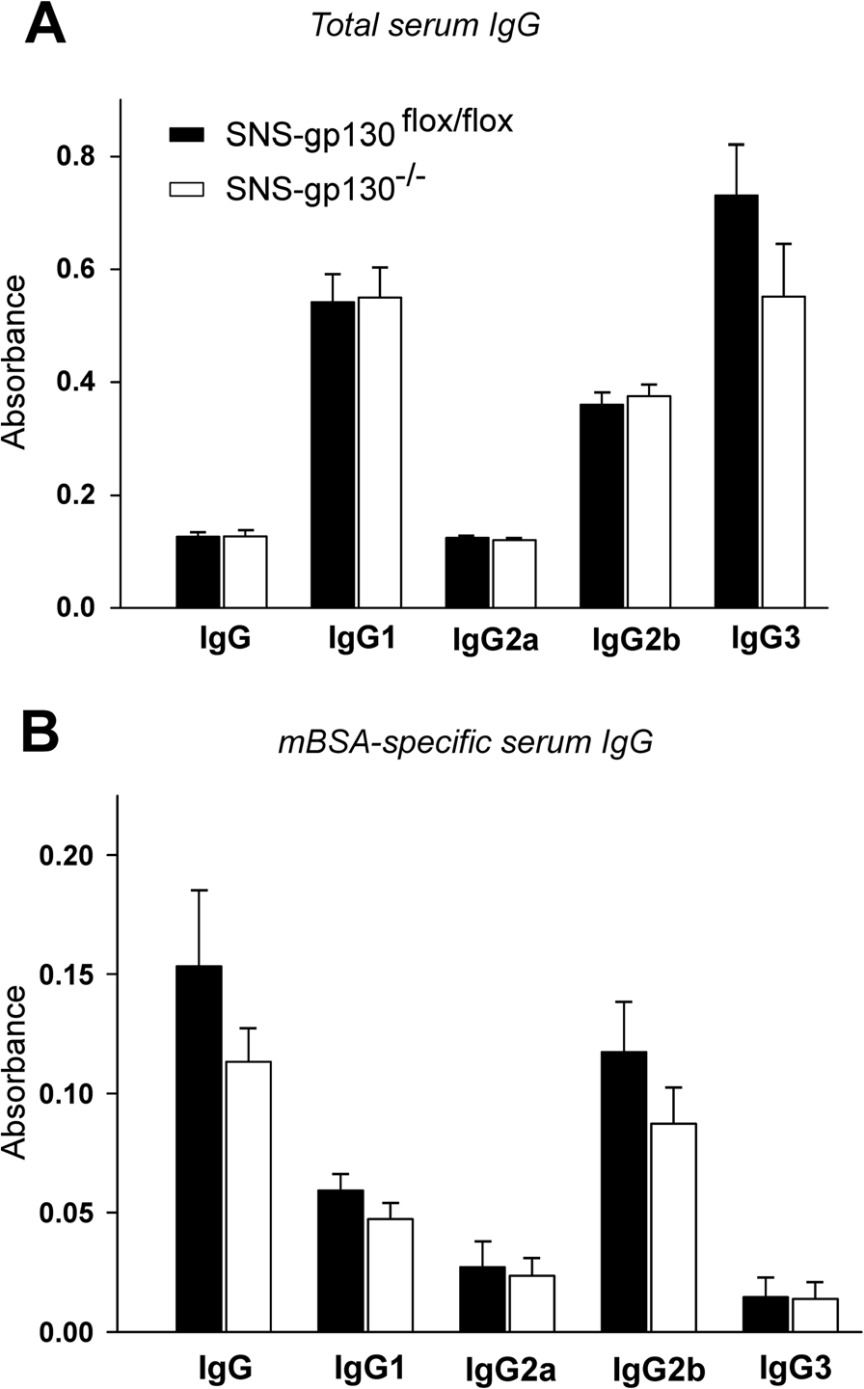
Serum immunoglobulin G (*IgG*) levels on day 3 of AIA. The amount of total serum IgG (**a**) as well as IgG specific for the antigen mBSA (**b**) do not show significant differences between both groups of mice. Values are mean ± SEM (*n* = 8–10 per group). *mBSA* methylated bovine serum albumin

Because we were particularly interested in the role of sensory neurons on the effect of IL-6 signaling in AIA, we measured the concentration of CGRP and IL-6 in the serum of SNS-gp130^−/−^ and SNS-gp130^flox/flox^ mice at day 3 of AIA (Fig. 3). Furthermore we assessed a number of immune parameters in SNS-gp130^−/−^ and SNS-gp130^flox/flox^ mice (Figs. 3, 4 and 5).

The serum of SNS-gp130^−/−^ mice contained less CGRP, IL-6, and IL-2 than the serum of SNS-gp130^flox/flox^ mice. The concentration of IFNγ was similar, the concentration of IL-17 in the serum was below the detection level (Fig. 3).

Furthermore, we measured the in vitro concentrations of a representative variety of T_H_1-, T_H_2-, and T_H_17-derived cytokines produced in lymph node and spleen cells. For this purpose we used single cell suspensions from inguinal, popliteal, subaortic lymph nodes and spleens, removed at day 3 of AIA, and stimulated them with either anti-CD3 antibodies for overall T cell receptor stimulation, or with mBSA for antigen-specific restimulation. No significant differences in the release of cytokines were observed between SNS-gp130^flox/flox^ and SNS-gp130^−/−^ mice upon unspecific stimulation of cells with anti-CD3 antibodies (Fig. 4a, b). Only the amount of IL-2 produced by spleen cells was decreased in SNS-gp130^−/−^ mice. However, upon antigen-specific restimulation with mBSA, the cells from SNS-gp130^−/−^ mice released significantly more IL-17 and IFNγ than cells from SNS-gp130^flox/flox^ mice (Fig. 4c, d).

No differences in the level of immunoglobulins in serum were detected between both groups of mice, neither for total immunoglobulins nor immunoglobulins specific for the antigen mBSA (Fig. 5a, b).

### CGRP and inflammation

**Fig. 6:**
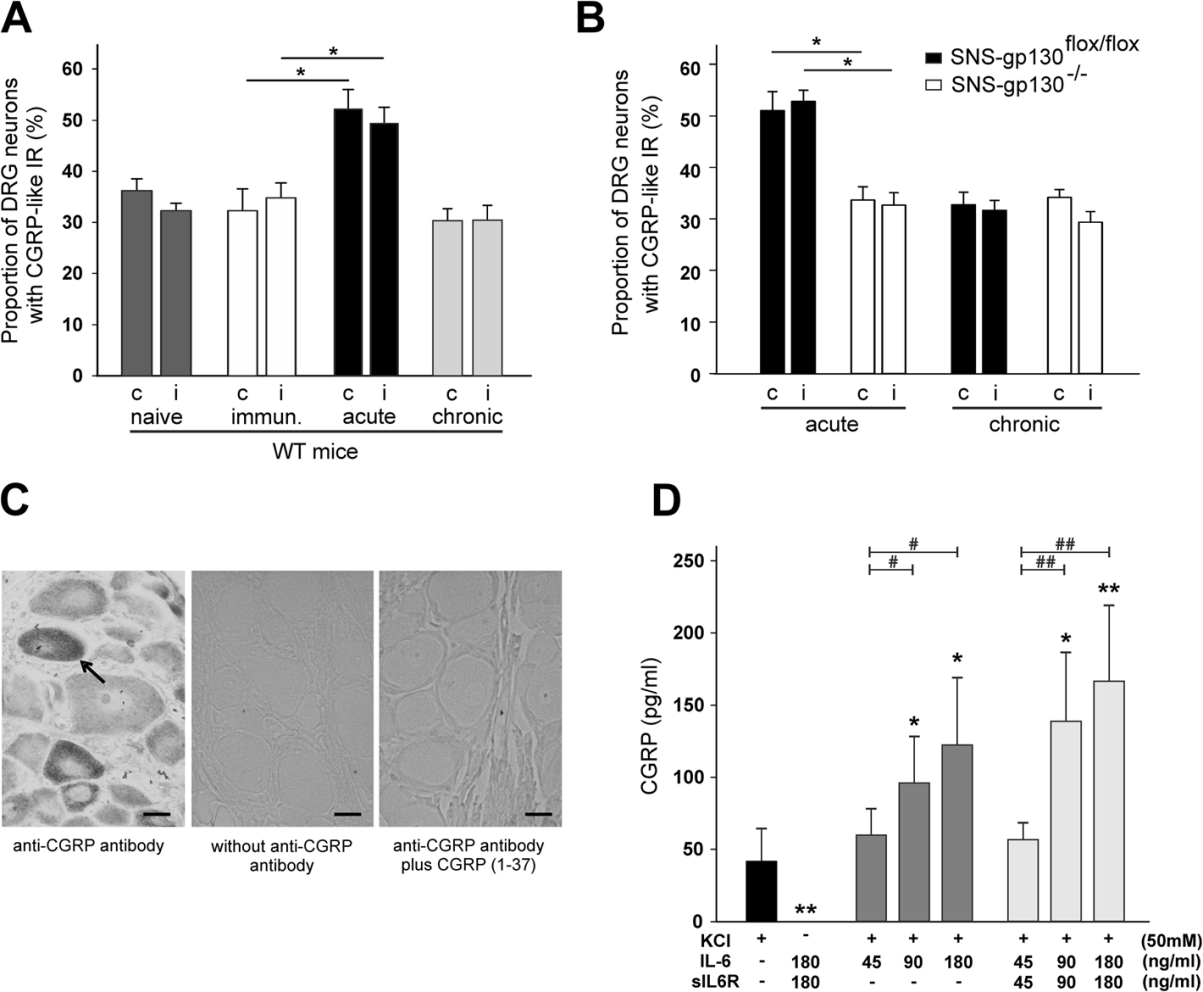
Upregulation of the expression of CGRP-like IR in vivo in DRG neurons and CGRP release in vitro from DRG neurons. **a**. Proportions of CGRP-positive DRG neurons in the contralateral (c) and ipsilateral (*i*, side of inflammation) lumbar DRGs in naïve control wild-type (*WT*) mice (*n* = 5), immunized WT mice (*n* = 4), WT mice at 3 days of AIA (acute, *n* = 4) and WT mice at 21 days of AIA (chronic, *n* = 4). **b**. Proportions of CGRP-positive DRG neurons in the contralateral and ipsilateral lumbar DRGs in SNS-gp130^flox/flox^ (*n* = 4–6) and SNS-gp130^−/−^ mice (*n* = 5) at 3 days of AIA (acute) and at day 12 of flare-up (chronic). Significant differences occur between SNS-gp130^flox/flox^ and SNS-gp130^−/−^ mice in the “acute phase”. Values are mean ± SEM. **c**. DRG section showing CGRP-positive neurons (see *arrow*) after incubation with anti-CGRP antibody (*left*). Control staining of DRG section without anti-CGRP antibody (*middle*) and with CGRP (1–37)-preincubation of the anti-CGRP antibody (*right*). Scale bar = 10 μm. **d**. CGRP release in vitro from DRG neurons from WT mice. Illustrated CGRP concentrations in the supernatants show the evoked release minus the basal production in the same time. *p < 0.05, **p < 0.01 significant differences referring to “only KCl”. ^#^ *p* < 0.05, ^##^ *p* < 0.01 significant differences between different concentrations of IL-6/sIL6R. Values are mean ± SD; *n* = 5 each. *CGRP* calcitonin gene-related peptide, *CGRP-like IR* CGRP-like immunoreactivity, *DRG* dorsal root ganglion, *IL-6* addition of IL-6 to the culture medium, *sIL6R* addition of sIL6R to the culture medium, *KCl* depolarization of neurons by addition of KCl (50 mmol/l) to the culture medium

The lower concentration of CGRP in the serum of SNS-gp130^−/−^ mice led us to investigate the role of CGRP in AIA. First, we quantified the proportions of CGRP-positive lumbar DRG neurons before and during the acute phase (day 3) and the chronic phase (day 21) of AIA. In WT mice we found a bilateral increase of the proportion of DRG neurons expressing CGRP at day 3 of AIA in comparison to the immunized control mice (Fig. 6a). Such an increase was not present any more at day 21 of AIA. At day 3 the SNS-gp130^flox/flox^ mice showed a similar bilateral upregulation of the CGRP-positive neurons as WT mice but in SNS-gp130^−/−^ mice this effect was not observed (Fig. 6b). CGRP-like immunoreactivity (CGRP-like IR) was expressed in small- and medium-sized DRG neurons (Fig. 6c). These data suggest a role of CGRP in the development of AIA, and they indicate that SNS-gp130^−/−^ and SNS-gp130^flox/flox^ mice differed in this aspect.

**Fig. 7:**
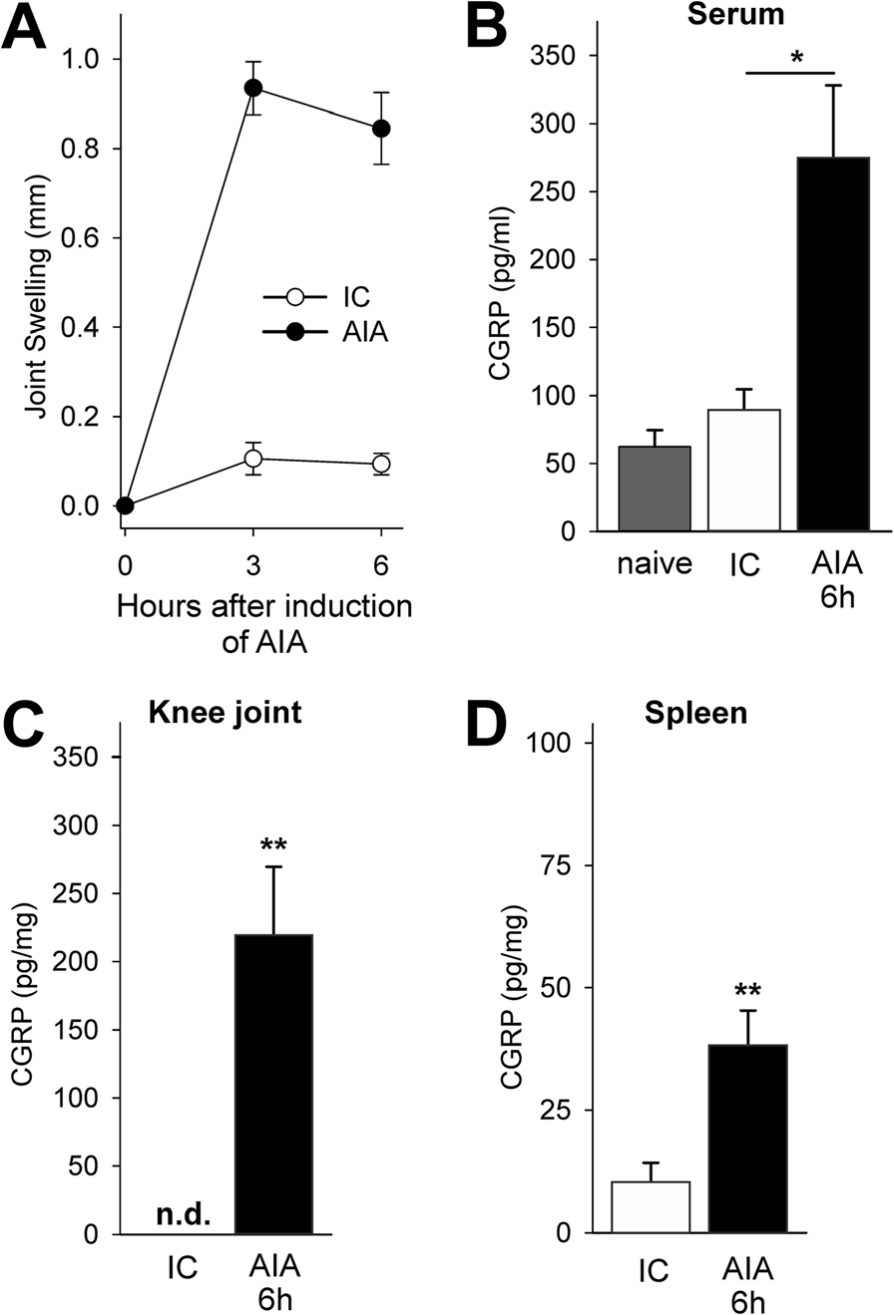
Increase of the CGRP concentration in the knee tissue, the serum and the spleen during beginning AIA. **a**. Significant swelling of the knee joint after induction of AIA. Immunized control mice (IC) received intraarticular NaCl instead of intraarticular mBSA. **b**-**d**. Enhanced concentration of CGRP in the serum, in the knee tissue, and in the spleen 6 h after induction of AIA (for knee joint and spleen CGRP concentration in relation to the total protein concentration). Values are mean ± SEM, *p < 0.05, **p < 0.01. *AIA* antigen-induced arthritis, *CGRP* calcitonin gene-related peptide, *IC* immunized mice which received intraarticular NaCl instead of mBSA, *n.d.* not detectable

Second, the injection of the antigen mBSA into the knee joint of immunized mice (induction of AIA) caused significant swelling of the knee joint within 3 h (Fig. 7a, AIA) whereas the intraarticular injection of NaCl in immunized mice did not cause swelling of the knee (Fig. 7a, IC). Within 6 h of AIA the CGRP concentration in the serum, in the knee tissue and in the spleen increased significantly (Fig. 7b-d).

Third, in order to test whether there is a link between IL-6 signaling and the release of CGRP, we tested whether IL-6 or IL-6/sIL6R complexes caused release of CGRP from sensory neurons (Fig. 6d). Supernatants from DRG neurons showed a basal release of CGRP (14.4 ± 10.1 pg/ml). Depolarization with KCl significantly increased the CGRP release (Fig. 6d, column 1), IL-6/sIL6R without KCl had no effect (column 2). However, either IL-6 alone or IL-6/sIL6R enhanced the release of CGRP by KCl (columns 3–8). Thus IL-6 signaling enhances the release of CGRP evoked by depolarization of the neurons.

### Cytokine expression profiles of lymphocytes from naive SNS-gp130^−/−^ and SNS-gp130^flox/flox^ mice

**Fig. 8:**
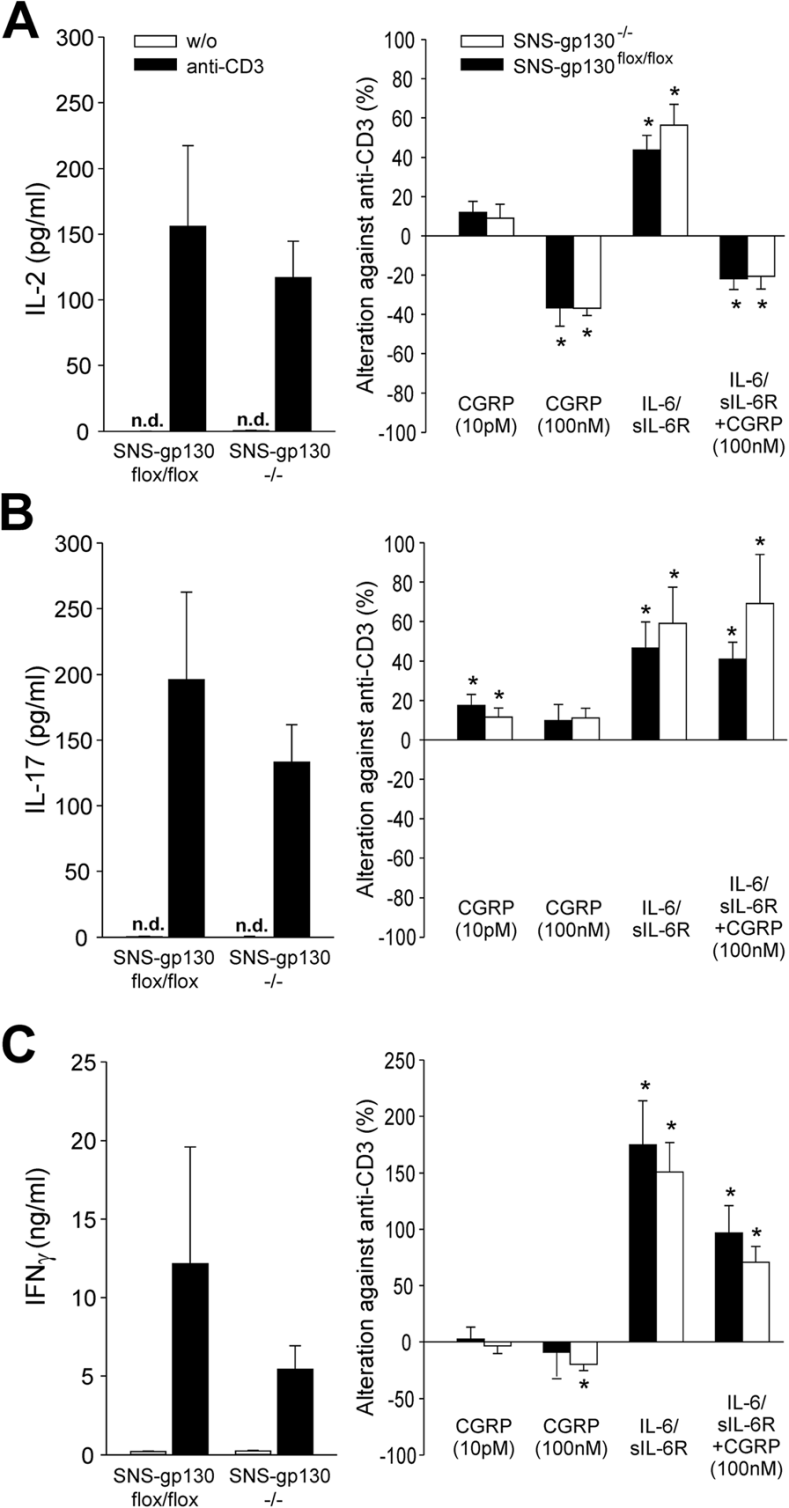
Cytokine expression profiles of lymphocytes from naïve SNS-gp130^flox/flox^ and SNS-gp130^−/−^ mice. Levels of (**a**) IL-2, (**b**) IL-17 and (**c**) IFNγ in supernatants of unstimulated (*w/o*) or anti-CD3-stimulated spleen cells. Additionally, cells were preincubated for 1 h with CGRP (100 nM or 10 pM) and/or IL-6/sIL-6R (90 ng/ml) before anti-CD3 stimulation. Values (mean ± SEM) are given as absolute concentrations (pg/ml or ng/ml) or as changes against cytokine concentration of anti-CD3 stimulation (baseline) in %. *p < 0.05 compared to baseline, by Wilcoxon’s matched-pairs signed-rank test. *n* = 6 per group. *CGRP* calcitonin gene-related peptide, *IFNγ* interferon-γ, *IL* interleukin *sIL-6R* soluble IL-6 receptor

In order to test whether lymphocytes from SNS-gp130^−/−^ and SNS-gp130^flox/flox^ mice show per se different reactions to activation, we stimulated lymphocytes with anti-CD3 and tested how either CGRP, IL-6/sIL-6R, or IL-6/sIL-6R plus CGRP influence the release of IL-2, IL-17, and IFNγ (Fig. 8). Both lymphocytes from SNS-gp130^−/−^ and SNS-gp130^flox/flox^ mice exhibited release of IL-2, IL-17, and IFNγ upon anti-CD3 stimulation, but the concentration of the cytokines in the supernatants was similar between genotypes (Fig. 8a, b, c, *columns on the left*). As expected, the secretion of IL-2 was significantly reduced by 100 nM CGRP (see [29]), significantly enhanced by IL-6/sIL-6R, and significantly reduced by IL-6/sIL-6R plus CGRP (Fig. 8a, *columns on the right*). By contrast, the release of IL-17 was slightly enhanced by 10 pM CGRP and strongly enhanced by IL-6/sIL-6R alone or by IL-6/sIL-6R in the presence of CGRP (Fig. 8b). The release of IFNγ was weakly inhibited by 100 nM CGRP in lymphocytes from SNS-gp130^−/−^ mice, but strongly enhanced by IL-6/sIL-6R, an effect, attenuated by CGRP (Fig. 8c). Together, these data show that both CGRP and IL-6 modified cytokine release with the strongest and opposite modulation of IL-2 secretion by CGRP and IL-6/sIL-6R. Furthermore, lymphocytes from SNS-gp130^−/−^ and SNS-gp130^flox/flox^ mice did not significantly differ concerning their reaction to anti-CD3, CGRP, and IL-6/sIL-6R stimulation.

## Discussion

The study revealed a significant reduction of swelling during the development of AIA in SNS-gp130^−/−^ mice in which gp130 signaling was specifically depleted in sensory neurons. Pain-related behavior was also reduced in SNS-gp130^−/−^ mice. The serum of SNS-gp130^−/−^ mice exhibited significantly lower concentrations of CGRP, IL-6, and IL-2 at day 3 of AIA than the serum of SNS-gp130^flox/flox^ mice. In wild-type and SNS-gp130^flox/flox^ mice but not in SNS-gp130^−/−^ mice the proportion of dorsal root ganglion neurons expressing CGRP was upregulated during the acute phase of AIA. CGRP was found to be significantly increased in the knee joint, serum and spleen during the first hours of AIA indicating the activation of CGRP-positive sensory neurons. In cultured DRG neurons CGRP release was evoked by IL-6 signaling. In naive lymphocytes from both SNS-gp130^flox/flox^ and SNS-gp130^−/−^ mice CGRP similarly reduced the secretion of IL-2, which may be responsible for the enhanced release of IL-17 and IFNγ upon antigen-specific restimulation. Compared to the lymphocytes of SNS-gp130^flox/flox^ mice, the lymphocytes from SNS-gp130^−/−^ mice showed an increased release of IL-17 and IFNγ upon antigen-specific restimulation. Taken together, the data show that gp130 signaling in sensory neurons is an important component in the development of immune-mediated arthritis.

In SNS-gp130^−/−^ mice the IL-6 signal transducer gp130 is only deleted in sensory neurons (mice that are systemically deficient for gp130 are embryonically lethal [30]). The reduction of swelling in SNS-gp130^−/−^ mice clearly indicates that sensory neurons are involved in the early development of inflammation and that IL-6 exerts part of its proinflammatory role by activating sensory neurons. The data further support that gp130 signaling contributes to inflammation-evoked mechanical hyperalgesia, corresponding to the sensitization of nociceptive sensory neurons for mechanical stimuli by IL-6 that has been previously reported for the complete Freund’s adjuvant and neuropathic pain models [13–15, 31]. During development of AIA SNS-gp130^flox/flox^ mice showed a decrease of the withdrawal threshold on the inflamed side and an increase of the threshold on the noninflamed side. The decrease of the threshold at the side of inflammation displays the secondary hyperalgesia at the paw. The increase of threshold at the noninflamed side (consistently observed in several studies on AIA in mice) is more difficult to explain. In order to withdraw the paw from a stimulus the mouse moves the knee joint. If the mouse withdraws the healthy leg, the inflamed leg is loaded and may cause discomfort. From rats with AIA in the knee joint in which static weight bearing can be measured we know that they put much less weight on the inflamed side than on the healthy side, i.e., they avoid to put load on the inflamed side. We believe, therefore, that the mouse tolerates a higher withdrawal threshold on the healthy side in order to prevent loading of the inflamed side. Therefore we propose the left-right difference as a measure of hyperalgesia. The left-right difference was significantly lower in SNS-gp130^−/−^ mice indicating less hyperalgesia in SNS-gp130^−/−^ mice than in SNS-gp130^flox/flox^ mice.

The main focus of the present study was on the role of sensory neurons and the IL-6 signaling of sensory neurons in the development of inflammation. Strikingly, swelling of the joint was reduced in the mice in which gp130 signaling was selectively deleted in sensory neurons, which display signatures of reduced excitability [32]. These data strongly suggest that sensory neurons contribute to the development of AIA by the mechanism of neurogenic inflammation. The latter process is largely, but not exclusively, linked to CGRP that is present in 30–40 % of small- and medium-sized DRG neurons supplying multiple tissues [33]. These peptidergic neurons rapidly release CGRP upon noxious stimulation [34] and during development of acute joint inflammation [1, 35]. CGRP is a potent vasodilator and associated with neurogenic inflammation [18]. Numerous sensory fibers containing CGRP are found in close proximity to the synovial capillary network [36] which produces the synovial fluid [37]. Here we found a strong CGRP release into the joint tissue and adjacent tissues in the first hours of AIA. In order to avoid further release of CGRP by dissecting procedures we used snap-frozen tissue blocks, which contained the knee joint and adjacent structures for the analysis of CGRP release. Therefore we cannot precisely differentiate how much the individual tissue types contributed to the CGRP release.

Similar to other models of inflammation [38, 39], we found a rapid upregulation of CGRP-like IR in lumbar DRG neurons during AIA, which is evident from the increase of the proportion of neurons expressing CGRP. Thus CGRP-expressing sensory neurons are markedly activated at the onset of AIA. Notably, the increase of the proportion of DRG neurons with upregulation of CGRP-like IR was observed bilaterally. Bilateral changes upon unilateral arthritis are common in the AIA model although the mechanism for such a bilateral effect has not been discovered [40].

Because CGRP is rapidly increased in the joint during AIA development, we believe that the activation of neurons by IL-6 and the CGRP release are linked. Several findings support this view. First, in isolated DRG neurons IL-6 signaling enhances the KCl (depolarization)-induced release of CGRP. The need of a costimulatory signal (here KCl) was also observed in other studies on IL-6-triggered CGRP release [41, 42] suggesting that IL-6 signaling alone is not sufficient to elicit action potentials. Second, the proportion of DRG neurons expressing CGRP-like IR was not upregulated in SNS-gp130^−/−^ mice whereas it was upregulated in SNS-gp130^flox/flox^ and WT mice. Other data established a direct link between IL-6 signaling and CGRP release. In sensory neurons, gp130 regulates the expression of the transient receptor potential ankyrin 1 (TRPA1) ion channel, which is relevant for mechanonociception, and in SNS-gp130^−/−^ mice TRPA1 is significantly less expressed [31]. Importantly, TRPA1 is also involved in the release of CGRP from sensory neurons [43]. The release of CGRP was also implicated in the generation of pain in arthritis models [44, 45] showing another link between IL-6 and CGRP.

In support for a role of CGRP and neuronal IL-6 signaling in the development of inflammation we found a reduction of the concentration of CGRP in the serum of SNS-gp130^−/−^ mice. Interestingly, the concentrations of IL-6 and IL-2 were also decreased. It is unclear why the concentration of these cytokines was reduced in SNS-gp130^−/−^ mice but the levels of CGRP, substance P and IL-6 are elevated in serum and synovial fluid of RA and osteoarthritis patients [19]. Thus the activation of sensory neurons by IL-6 may also regulate the concentration of IL-6. For example, monocytes, the major source of IL-6 in serum, and fibroblasts are activated by CGRP [46, 47]. IL-6 can also be produced in a proportion of sensory neurons themselves [48–50]. This was not further explored.

Interestingly, histopathology did not identify a significant difference of AIA in SNS-gp130^−/−^ and SNS-gp130^flox/flox^ mice, neither at the acute nor at the chronic stage. However, histopathology mainly assesses the cellular joint infiltration that is driven by cytokines, and not the edema [51]. Notably, in SNS-gp130^flox/flox^ mice the swelling and the cellular infiltration was highly correlated but such a correlation was lost in the SNS-gp130^−/−^ mice, i.e., they had a strong cellular reaction in spite of reduced swelling. This led to the question whether SNS-gp130^−/−^ and SNS-gp130^flox/flox^ mice show differences in the immune responsiveness. The amounts of immunoglobulin in the serum were not different, but the spleen and lymph node cell cultures from SNS-gp130^−/−^ mice released more IL-17 and IFNγ after antigen-specific restimulation. In particular IL-17 is thought to be a pivotal driving force of autoimmune inflammation in animal models of arthritis as well as in human rheumatoid arthritis [52, 53].

A possible explanation for this observation could be that T cells express receptors for CGRP [54, 55], that CGRP-positive nerve fibers immunoreactive for CGRP were detected in spleen and lymph nodes of mice [54, 56], and that CGRP inhibits antigen-specific T cell proliferation [55] and accumulation of IFNγ mRNA [29]. Thus the loss of IL-6 signaling in sensory neurons and the lack of IL-6-induced CGRP secretion may lead to an enhanced release of IL-17 and IFNγ in lymphocytes of SNS-gp130^−/−^ mice. However, neither in naive lymphocytes from SNS-gp130^−/−^ mice nor from SNS-gp130^flox/flox^ mice CGRP reduced the release of IL-17 or IFNγ upon anti-CD3 stimulation. By contrast, as described previously [29], CGRP at an effective dose reduced the release of IL-2. This cytokine constrains the T helper 17 cell generation by STAT5 and reduces the synthesis of IL-17 and IFNγ in lymphocytes [57–59] thus opposing the stimulating effect of IL-6 on lymphocytes (see Fig. 8). The reduction of serum concentration of IL-2 in SNS-gp130^−/−^ mice may attenuate the inhibitory effect of IL-2, and this may allow a stronger release of IL-17 and IFNγ in lymphocytes of SNS-gp130^−/−^ mice upon antigen-specific restimulation.

## Conclusions

The proinflammatory effect of IL-6 in immune-mediated arthritis depends in part on the effect of IL-6 on sensory neurons because the selective deletion of IL-6 signaling in sensory neurons significantly reduces the inflammatory swelling in the afflicted joint. IL-6 enhances the release of CGRP evoked by depolarization, a neuropeptide with strong vasodilator activity, from sensory neurons thus activating neurogenic mechanisms of inflammation. In addition, neuronal IL-6 signaling might influence the systemic cellular inflammatory response because lymphocytes from mice without neuronal IL-6 signaling show enhanced release of IL-17 and IFNγ upon antigen-specific restimulation. Together the present data indicate a role of neuronal IL-6 signaling in the coordination of the whole arthritic process and not just in the generation of arthritic pain. Rather IL-6 signaling functionally connects the nervous and immune system in a bidirectional way.

## Abbreviations

AIA: antigen-induced arthritis
AUC: area under the curve
CGRP: calcitonin gene-related peptide
CGRP-like IR: CGRP-like immunoreactivity
DRG: dorsal root ganglion
ELISA: enzyme-linked immunosorbent assay
gp130: glycoprotein 130 (transmembrane signal-transducing subunit)
H&E: hematoxylin and eosin
IFNγ: interferon-γ
IgG: immunoglobulin
IL: interleukin
IL-6R: IL-6 receptor
mBSA: methylated bovine serum albumin
Na_v_1.8: voltage-gated sodium current 1.8
sIL-6R: soluble IL-6 receptor
SNS: sensory neuron specific
TRPA1 ion channel: transient receptor potential ankyrin 1 ion channel
WT: wild-type
